# Respiratory Diagnostic Tools in Neuromuscular Disease

**DOI:** 10.3390/children5060078

**Published:** 2018-06-15

**Authors:** Jackie Chiang, Kevan Mehta, Reshma Amin

**Affiliations:** 1 Holland Bloorview Kids Rehabilitation Hospital, The University of Toronto, Toronto, ON M4G 1R8, Canada; jackie.chiang@sickkids.ca; 2 Division of Respiratory Medicine, 4539 Hill Wing, The Hospital for Sick Children, 555 University Avenue, Toronto, ON M5G 1X8, Canada; kevan.mehta@sickkids.ca

**Keywords:** children, neuromuscular disease, pulmonary function testing, respiratory muscle testing, peak cough flow, oximetry, capnography, polysomnogram

## Abstract

Children with neuromuscular disease (NMD) are at risk of acquiring respiratory complications. Both clinical assessments and respiratory diagnostic tests are important to optimize the respiratory health and care of such children. The following respiratory diagnostic tools and their utility for evaluating children with NMD are discussed in this article: lung function testing (spirometry and lung volumes), peak cough flow (PCF), respiratory muscle strength testing, oximetry, capnography, and polysomnography.

## 1. Introduction

Children with neuromuscular disease (NMD) are at risk of developing respiratory complications [1]. Muscle weakness can lead to ineffective cough and retained secretions, which predispose children to recurrent pneumonias and atelectasis [2]. This may result in decreased lung compliance, increased airway resistance, and heightened ventilatory demands, eventually leading to respiratory insufficiency, first during sleep and subsequently progressing to diurnal hypoventilation [2].

A thorough clinical evaluation of respiratory health is important for all children with NMD. However, clinical assessments in isolation have been found to be a poor predictor of both sleep-disordered breathing (SDB) and survival in children with Duchenne muscular dystrophy (DMD), the most common type of NMD in children [3]. Thus, objective respiratory diagnostic tests are crucial and can be used to help to evaluate the respiratory status at the time of diagnosis, monitor disease, carry out informed decision-making regarding surgical interventions, and potentially help to determine prognosis [4]. The following respiratory diagnostic tools and their utility for evaluating children with NMD are discussed in further detail in this article: lung function testing (spirometry and lung volumes), peak cough flow (PCF), respiratory muscle strength testing, oximetry, capnography, and polysomnography. Given the extensive scope of diagnostic imaging for children with NMD, a discussion of these tests is not covered in this review.

## 2. Clinical Assessment

A clinical evaluation of respiratory health should be included in every medical consultation for children with NMD [3]. A thorough history on the frequency, severity, and treatment of respiratory infections should be obtained, as well as the status of comorbid conditions including scoliosis and cardiac complications. Symptoms suggestive of sleep disturbances should be screened for, including snoring, pauses in breathing, morning headaches, and daytime somnolence [3]. Depending on the type of NMD, an assessment of muscle weakness and fatigability, change in voice or cough strength, sialorrhea or secretion management problems, and dysphagia or choking may be relevant. Spot-check assessments of oxygen saturation (SpO_2_) and carbon dioxide (end-tidal CO_2_ or pCO_2_) also aid in evaluating baseline respiratory status. In children with DMD experiencing symptoms and signs consistent with a respiratory infection, a persistent SpO_2_ < 95% in room air warrants antibiotic therapy [5]. In addition, daytime hypoxia (SpO_2_ < 95%) and hypercapnia (end-tidal CO_2_ or pCO_2_ > 45 mmHg) is indicative of progressive muscle weakness and is preceded by nocturnal hypoventilation necessitating surveillance sleep evaluations [3,5]. Supplemental oxygen for hypoxemia should be used with caution in children with NMD because of potential impairment of the central respiratory drive, resulting in further exacerbation of hypercapnia [5]. Hypoxemia related to hypoventilation should be supported with ventilator support first and with oxygen therapy as a secondary adjunct if required [5].

## 3. Evaluation of Lung Function

### 3.1. Spirometry

Spirometry is an important component of routine pulmonary function testing that is often used as a screening test for general respiratory health [6]. Spirometry involves measuring the inhalation and exhalation volumes of air as a function of time via a spirometer [6]. In order for the results of spirometry to be deemed acceptable and reliable, several criteria need to be met, including spirograms that are free from artefacts, have good starts, and show satisfactory exhalation (duration of ≥6 or ≥3 s in children or a plateau in the volume-time curve) [6]. After three spirograms have been obtained, the two largest values of Forced Expiratory Volume in 1 s (FEV_1_; volume of air exhaled forcefully in the first second after a maximal inspiration to total lung capacity ) must be within 0.15 L of each other, and the two largest values of Forced Vital Capacity (FVC; total volume of air forcefully expelled after a maximal inspiration to total lung capacity) must be within 0.15 L of each other [6]. Data is typically reported in absolute litres as well as in percent predicted values on the basis of identified reference ranges that take the age, gender, and height of the child into account [6]. On average, spirometry that is acceptable and reproducible according to the American Thoracic Society lung function criteria can generally be performed on children 6 years of age and above [6]. The Global Lung Health Initiative has developed spirometry reference ranges for essentially all ages (range of 4–80 years) [7]. However, cognitive or physical limitations in some children with NMD may delay or circumvent the ability to perform spirometry. It is important to note that standing height may be unreliable or unattainable in children with NMD (e.g., may not be ambulatory or may have significant scoliosis), and therefore surrogate measures such as arm span or ulna length should be used, with the latter potentially being easier to measure in the context of significant contractures [3]. In addition, although spirometry is typically performed via a mouthpiece, individuals with NMD may be unable to provide a suitable lip seal around a conventional mouthpiece and thus a flanged mouthpiece or face mask should be considered [3].

Both FEV_1_ and FVC are particularly pertinent in individuals with NMD. They are often reduced in children with NMD compared to healthy controls because they are determined by inspiratory and expiratory muscle strength as well as by chest wall and lung compliance. Total lung capacity itself is reduced because of reduced muscle strength, and as a result, there is a reduction in the exhalation airflow despite structurally normal airways. The volume of forcefully exhaled air in 1 s (FEV_1_) is reduced in proportion to FVC. Therefore, the FEV_1_/FVC ratio generally remains in the normal range (i.e., 80–100%) or may even be high [1]. This constellation of spirometry findings is categorized as being consistent with restrictive lung disease. Despite FEV_1_ and FVC both being consistently reduced in NMD patients, the severity of any spirometric abnormality (whether restrictive or obstructive in nature) is still based on FEV_1_ alone, whereby mild is >70% predicted; moderate is 60–69% predicted; moderately severe is 50–59% predicted; severe is 35–49% predicted; and very severe is <35% predicted [8].

In contrast to FVC, vital capacity (VC) does not require a forceful manoeuvre but still records the maximum amount of air an individual can expel from the lungs after a maximum inhalation. Even children with NMD who have difficulty performing a forceful manoeuvre can still often be reliably measured using a slow VC manoeuvre [6]. In fact, VC should be measured in all patients with NMD as part of the respiratory assessment [3]. It may be particularly helpful in monitoring disease progression, as it was shown to strongly correlate with the number of chest infections and days of antibiotic treatment in the preceding year in one pediatric study on a mixed group of NMD conditions [9]. It may also aid in determining if diaphragmatic weakness exists, which is suggested when a decrease of 25% or more in VC values is noted when spirometry is performed supine as compared to in the upright position [9].

In children with DMD, specific recommendations have been put forth with respect to the timing of pulmonary function testing. Spirometry is recommended early in the course of disease (around 6 years of age), during every clinic visit and before surgery [5,10]. The relationship between the absolute value of FVC and age in individuals with DMD has classically been divided into three categories: (1) gradual increase during the early years when still ambulatory; (2) plateau phase between 10 and 12 years of age when ambulation is lost; (3) gradual but persistent decline during adolescence and adulthood [11]. However, with the initiation of corticosteroid therapies such as deflazacort, this timeline has shifted towards a longer preservation of ambulation and improved median survival than previously described [12]. Nevertheless, an FVC of <1 L still remains the best negative predictor of survival in individuals with DMD [10]. In addition, for those undergoing surgical procedures, preoperative training in and postoperative use of non-invasive ventilation should strongly be considered for patients with a baseline FVC of <50% predicted and are essential for patients with FVC of <30% predicted [5].

### 3.2. Lung Volumes

Measurements of fractional lung volumes (i.e., volume of gas within the lungs) can be taken using specialized techniques such as body plethysmography, gas dilution, or washout [13]. Lung volumes are related to body size, with height as the most important correlating variable [8]. Similarly to spirometry, lung volume measurements are usually attainable by 6 years of age. Total lung capacity (total volume of air in the lungs) is determined by the strength of contraction of chest muscles as well as by the lung’s inward recoil [4]. Individuals with muscle weakness often display a reduced TLC, thereby demonstrating a restrictive ventilatory pattern. A restrictive defect is characterized by a reduction in TLC below the fifth percentile of the predicted value and by a normal FEV_1_/VC [8]. The severity of the restriction can be determined using percentage predicted values for TLC with ≥80% predicted as normal, 70–80% predicted as mild restriction, 60–70% predicted as moderate restriction, and less than 60% predicted as severe restriction [4].

Total lung capacity is comprised of VC and residual volume (RV). Volume capacity is typically low as a result of loss of inspiratory muscle strength in addition to a decrease in lung compliance (e.g., diffuse microatelectasis) and chest wall compliance (e.g., scoliosis) [14]. Residual volume is the volume of air that remains in the lung after maximal expiration and is determined by the ability of the expiratory muscles to compress the chest wall inward. It is thus often elevated (or at least preserved) as a result of weakened expiratory muscles being unable to move the chest wall inward to completely deflate the lungs [14]. Residual volume may be one of the only abnormal findings early on in the course of the disease [4]. The increase in RV can be further exacerbated in those who develop scoliosis [1]. On the other hand, functional residual capacity (FRC; volume of gas present in the lung at end-expiration during normal tidal breathing) is often used to calculate other lung volumes and is typically found to be in the normal range (see Table 1).

## 4. Peak Cough Flow

Coughing is an important respiratory defense mechanism that clears potential pathogens and maintains airway patency. A typical cough consists of the following sequential steps: (1) a full inspiration; (2) closure of the glottis; (3) contraction of abdominal and expiratory respiratory muscles to generate a positive intrathoracic pressure; (4) opening of the glottis to allow forceful expulsion of air outward [1].

Children with NMD have a weak cough as a result of absent or reduced respiratory muscle strength. In addition, those with associated bulbar dysfunction may also have a weakened cough because of the inability to rapidly open the glottis and maintain patency of the upper airway during coughing [15]. An impaired cough results in retained secretions predisposing individuals to bronchial mucous plugging, which can then lead to microatelectasis, causing hypoxemia and hypercapnia [16]. Furthermore, it can increase the risk of respiratory infections over time, leading to a persistent inflammatory response and chronic lung damage [17].

**Figure 1:**
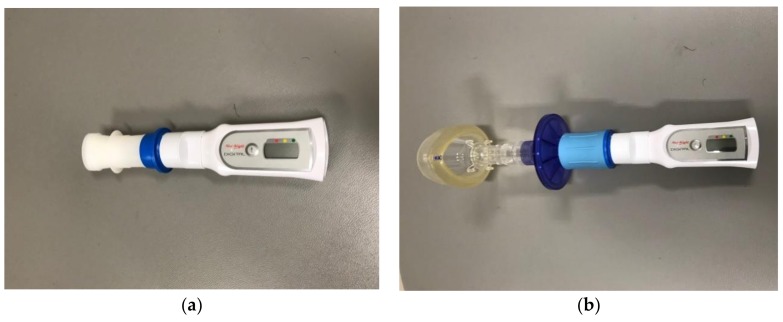
Peak flow meter used to measure peak cough flow in children with neuromuscular disease (NMD): (**a**) with mouthpiece; (**b**) with mask.

The strength of a cough can objectively be assessed in cooperative children starting at around 6 years of age when spirometry is able to be performed. It is assessed using a handheld peak flow meter (see Figure 1). The child inspires to TLC and then forcibly coughs into the device via a mouthpiece or mask while wearing nose clips. Normal PCF values for adults are greater than 400 L/min, whereas PCF values of 270 L/min or less have been shown to increase the risk of pneumonia in adults with NMD [18].

In children, PCF rates have been shown to vary according to gender, height, and body mass surface [19]. It has been recommended that PCF be used as part of the routine assessment of effective secretion clearance in children with NMD who are 12 years of age and older [3]. A cutoff of <270 L/min indicating a weak cough is only relevant for children 12 years of age or older, as for younger children, PCF values below this threshold may fall within the normal range [3]. Children with NMD and ineffective cough (including children over 12 years of age with PCF of <270 L/min) should be taught how to use cough augmentation techniques and devices, particularly if they have respiratory infections causing deterioration (see Table 2) [3]. Adolescent and adult patients with DMD have been recommended to use cough augmentation devices, such as the mechanical in-exsufflation (MIE) machine or lung volume recruitment (LVR) bag, once PCF values reach <270 L/min [5,10]. Mechanical in-exsufflation clears respiratory secretions by applying a positive pressure followed by a rapid shift to negative pressure, thereby simulating a natural cough. Lung volume recruitment consists of using a self-inflating resuscitation bag attached to a one-way valve. Compression of the LVR bag is coordinated with the patient’s sequential inhalations with the aim of hyperinflating the lungs, resulting in greater PCFs [20]. Unfortunately, although standard pediatric values for PCF have been published, thresholds predictive of respiratory complications in young children are not available [21].

## 5. Respiratory Muscle Strength Testing

**Figure 2:**
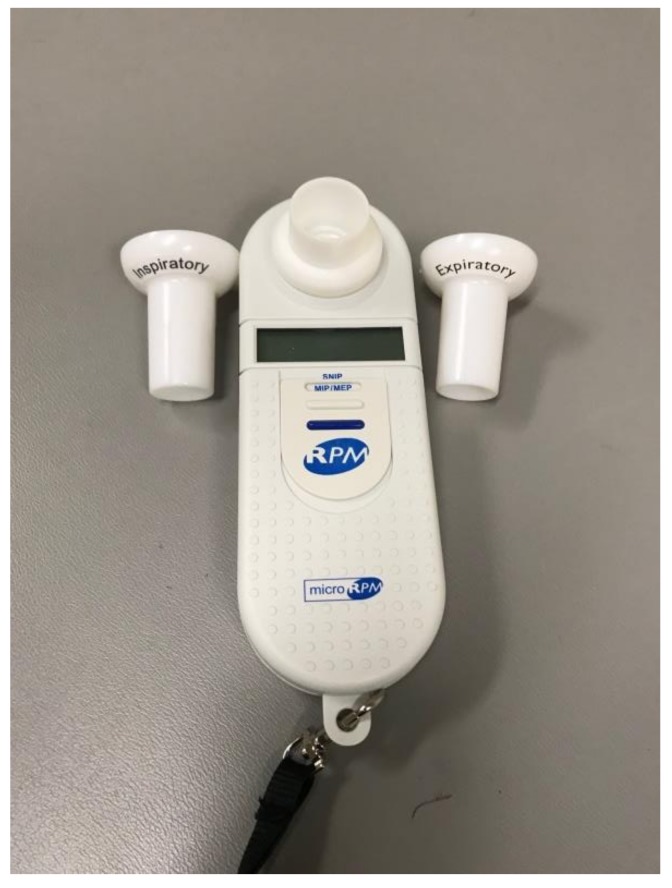
Maximal inspiratory pressure (MIP) and maximal expiratory pressure (MEP) device.

**Figure 3:**
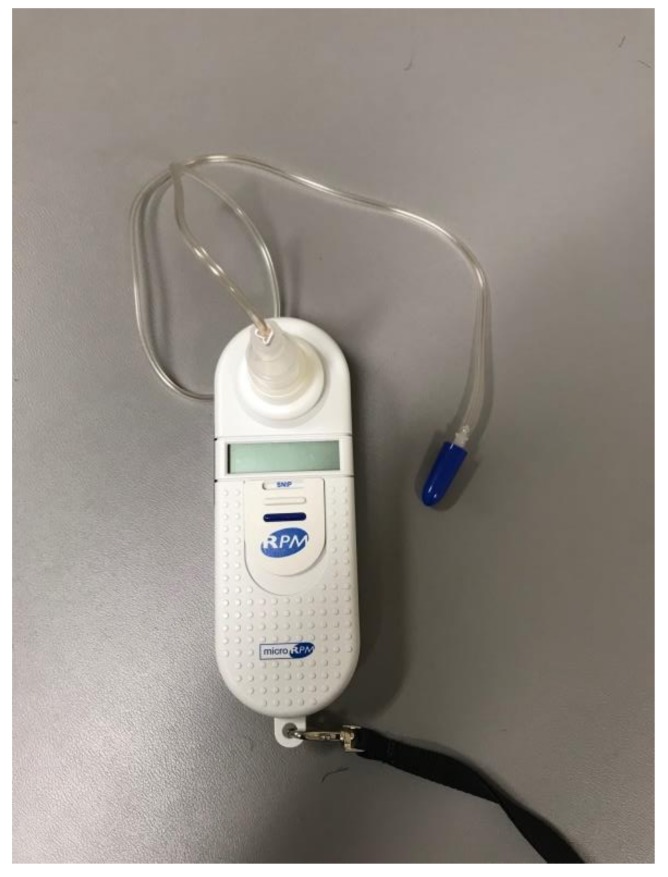
Sniff nasal inspiratory pressure (SNIP) device.

In addition to routine lung function testing (i.e., spirometry and lung volumes), respiratory muscle testing is recommended in children with NMD [21]. The most common respiratory muscle tests performed in children are those for maximal static pressures (inspiratory and expiratory; see Figure 2) and sniff nasal inspiratory pressure (SNIP; see Figure 3).

Maximal inspiratory pressure (MIP) is measured at the mouth; the child is seated and breathing through the mouthpiece and is then asked to forcefully inspire from RV [22]. Maximal expiratory pressure (MEP) is conversely measured from TLC [22]. In children, MIPs and MEPs increase with age and are generally greater in males than in females, even prior to puberty [23].

### 5.1. Maximal Expiratory Pressure

Maximal expiratory pressure has been shown to correlate well with VC and may in fact be an earlier marker for the progression of muscle weakness as it was noted to decline earlier than MIP and VC in a cross-sectional study of individuals, including children, with DMD [24]. Additionally, as for VC, it has been shown to correlate with the frequency of infections and days of antibiotic use in the preceding year [9]. Finally, MEP also appears to correlate with cough strength, whereby a MEP of <60 cm H_2_O indicates an ineffective cough, resulting in a recommendation to initiate cough augmentation techniques and use devices (see Table 2) [10].

### 5.2. Maximal Inspiratory Pressure

Maximal inspiratory pressure has additionally been shown to be highly correlated with VC for children with NMD and thus may also be helpful in predicting hypoventilation in such individuals [3]. In one cross-sectional study of pediatric patients with muscle disease in Germany, MIP of <4.0 kPa (or <40.8 cmH_2_O) was associated with SDB, and MIP of <2.5 kPa (or <25.5 cmH_2_O) was associated with nocturnal hypoventilation [25]. Unfortunately, existing pediatric reference ranges for normative values of MIPs and MEPs are limited to small, single-center cohorts [26,27,28].

### 5.3. Sniff Nasal Inspiratory Pressure

The measurement of SNIP is another non-invasive method to assess inspiratory muscle strength in children with NMD [21]. It consists of measuring, from FRC, the nasal pressure in an occluded nostril as the patient sniffs through the contralateral unobstructed nostril [23]. This pressure in the obstructed nostril reflects the pressure in the nasopharynx, which is reasonably indicative of alveolar pressure [22]. Values in healthy children between 6 and 17 years of age are similar to those found in adults, with a mean SNIP of 104 ± 26 cmH_2_O in males and 93 ± 23 cmH_2_O in females [26]. The main limitation of SNIP is the potential to underestimate inspiratory muscle strength when nasal obstruction exists (e.g., adenoids or nasal polyps) or in those with severe respiratory muscle weakness [21]. However, it has been shown to be correlated well with and easier to measure than MIP in patients with NMD [26]. SNIP also reportedly correlated well with FVC in a study of children with NMD, and whereas FVC measurements were obtained for only 25 of 41 patients, SNIP measurements were obtained for all children, suggesting that SNIP manoeuvres were also easier to perform than FVC and therefore may be a useful tool in evaluating the respiratory status in individuals with NMD, particularly in younger children who may not be able to perform other lung function tests [29].

## 6. Evaluation of Breathing during Sleep

Individuals with NMD are at risk of SDB. Sleep-disordered breathing is a broad condition that includes abnormalities in respiratory breathing pattern, gas exchange, and sleep architecture during sleep. SDB can be defined as the presence of (1) nocturnal hypoventilation, (2) obstructive sleep apnea (OSA), and/or (3) central sleep apnea (CSA) [30]. The definition of nocturnal hypoventilation in pediatric patients has been debated, as limited studies exist linking secondary end-organ damage to specific levels of CO_2_ measurements in children. The most commonly utilized definition in children arises from the American Academy of Sleep Medicine (AASM), which defines it as pCO_2_ of >50 mmHg for 25% or more of total sleep time during a polysomnogram (PSG) [31]. Obstructive events are defined as partial (hypopnea) or complete (apnea) airway closure resulting in sleep fragmentation and/or gas-exchange abnormalities [32]. The same disruption occurs in CSA but is a result of cessation (apnea) or shallow breaths (hypopnea) being taken, without evidence of upper airway obstruction [29].

Children with NMD may be at risk for all three types of SDB depending on their specific NMD diagnosis. For example, children with congenital myotonic dystrophy have a higher risk of CSA, while those with DMD may have OSA and/or nocturnal hypoventilation. Nocturnal hypoventilation is a result of inadequate minute ventilation during sleep that is typically secondary to reduced muscle strength; other factors can also contribute to this, such as scoliosis and obesity [1]. Weakness of upper airway muscles increases upper airway collapsibility causing obstruction, while reduced intercostal and diaphragm strength can lead to shallow breathing characteristic of central events [3]. Additionally, this respiratory instability further predisposes such children to central events.

The gold standard test to diagnose SDB in children is a PSG. However, other tests, including oximetry and capnography, are also used in clinical practice worldwide, given the often-limited access to PSG. Each of these diagnostic tools as well as their advantages and disadvantages are discussed in detail below.

### 6.1. Oximetry

Oximetry is a continuous recording of oxygen saturation using an oxygen probe attached most commonly to a digit, although the ear and forehead can also be used. Overnight oximetry has been commonly used as a screening tool for SDB in children with NMD.

It is important to be aware of the calibration metrics used for oximetry recordings when interpreting an oximetry study. In general, the device should be programmed to record data at least 60 times per second and averaged over 4–10 s, with data displayed every second, for adequate sensitivity [3]. In most PSG laboratories, the averaging time of oximeters is 3 s, thereby making oximetry in the laboratory more sensitive for detecting desaturations. The data also should be displayed in a format that allows for several hours of oximetry data to be viewed on a single screen; viewing the plethysmographic waveform may help in discriminating between a true oxygen desaturation and artifacts [3].

**Figure 4:**
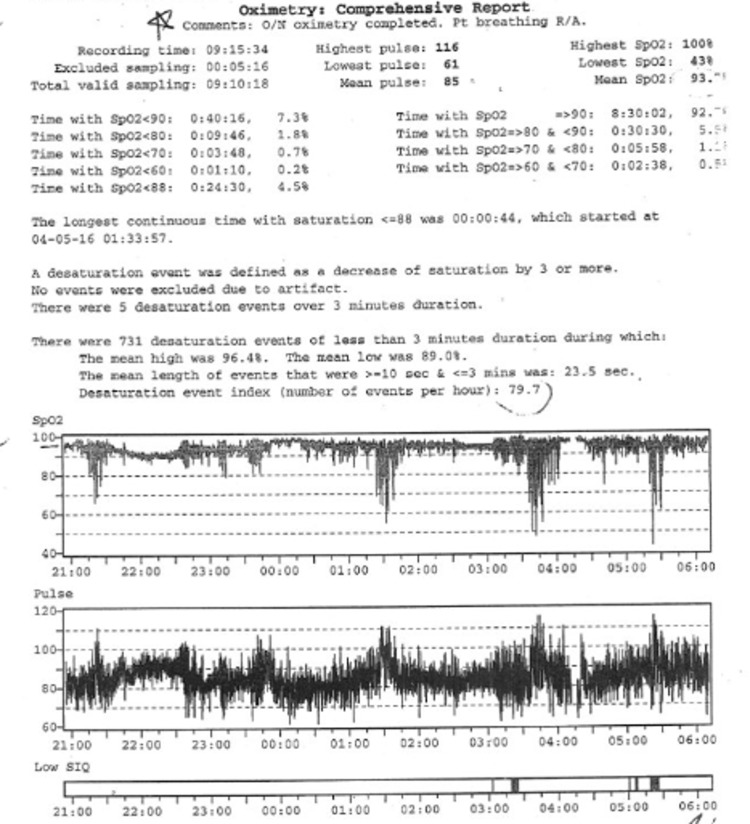
Noninvasive adjuncts used to measure CO_2_ levels during overnight polysomnograms. (**a**) Transcutaneous CO_2_ probe, which is applied to the skin; (**b**) nasal cannulae, which is inserted in the nares to measure end-tidal CO_2_ levels.

**Figure 5:**
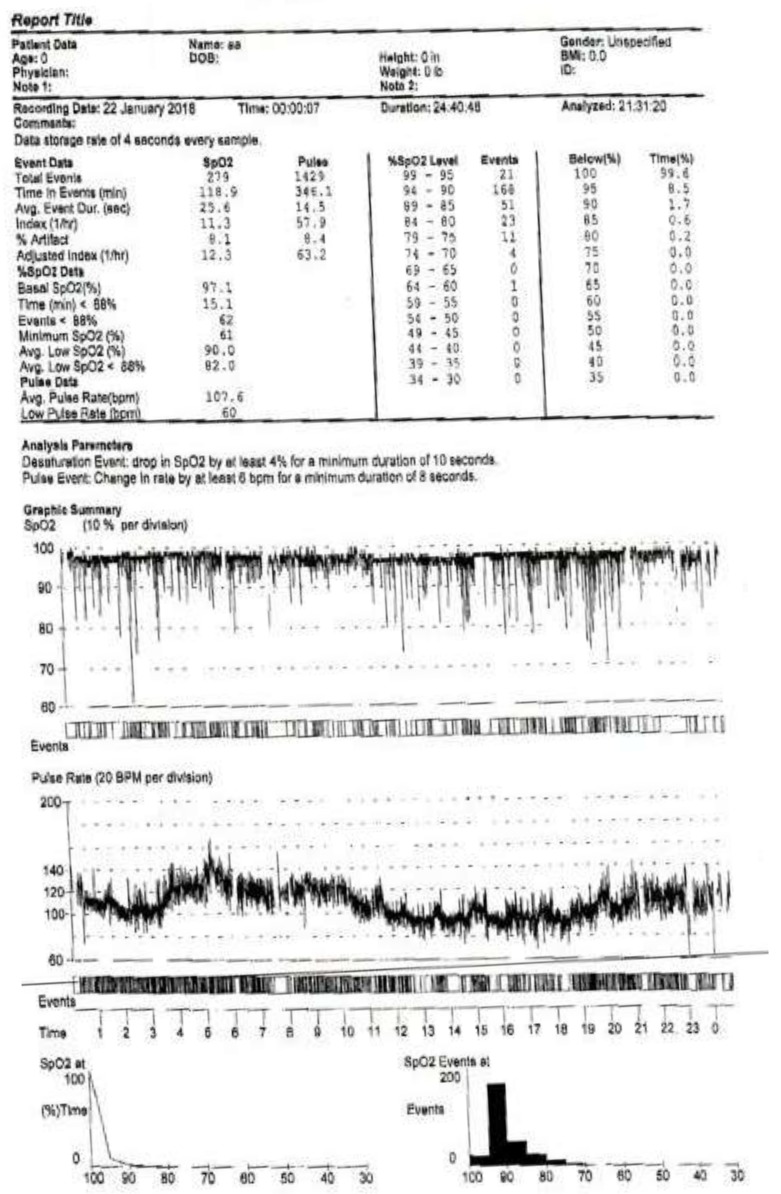
Overnight oximetry demonstrating the characteristic “sawtooth pattern” of oxygen desaturations suggestive of obstructive sleep apnea.

**Figure 6:**
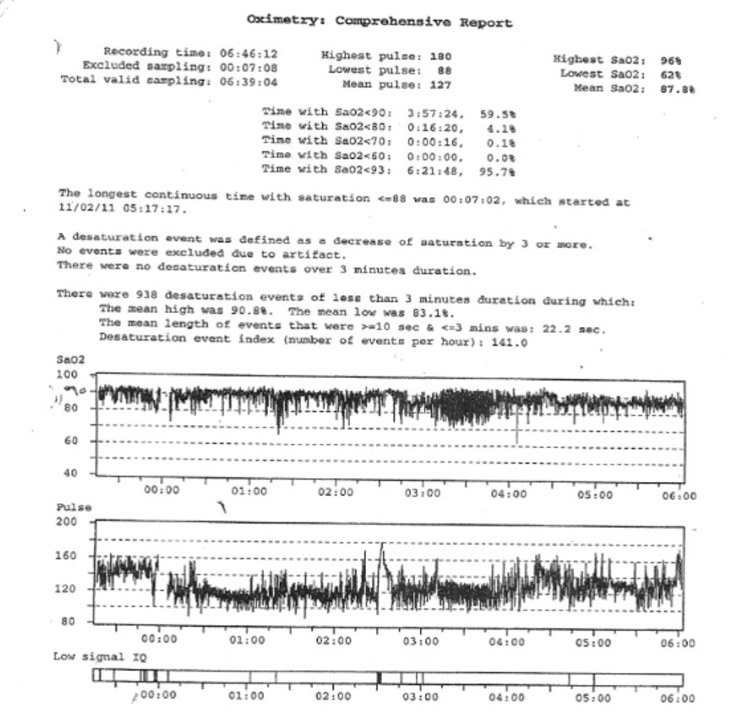
Overnight oximetry demonstrating low baseline oxygen saturation and frequent oxygen desaturations suggestive of hypoventilation.

Oxygen desaturation in overnight oximetry can be a consequence of OSA, CSA, and/or hypoventilation. In those who have oxygen desaturations associated with their SDB, the pattern of oxygen desaturation can be helpful in this regard, and the desaturation index can be a proxy measurement for severity. The desaturation index is defined as the number of desaturations over the recording divided by the number of hours of the recording (units are desaturations per hour). In children with OSA, the oximetry may show a characteristic sawtooth pattern of repeated cyclical desaturation during rapid eye movement (REM) sleep (see Figure 4) [3]. Central sleep apnea is usually associated with repeated oxygen desaturations throughout all stages of sleep (see Figure 5) [33]. In contrast, in the setting of significant nocturnal hypoventilation, the baseline oxygen saturations are low and/or there may be sustained periods of oxygen desaturations (see Figure 6). However, these characteristic patterns are not always present, and thus discerning types of respiratory events may be difficult, rendering oximetry often unreliable in establishing a definitive diagnosis of SDB, particularly in milder cases, when early intervention may be beneficial. However, technically adequate oximetry alone has been deemed an acceptable screening method for significant hypoventilation when CO_2_ monitoring and PSG are unavailable, on the basis that desaturations below 93% are commonly present in those with significant hypoventilation [3]. Furthermore, oximetry is advantageous in that it is less bothersome to the patient (only a single probe needs to be worn overnight) and is readily available, even being able to be performed at home.

### 6.2. Capnography

**Figure 7:**
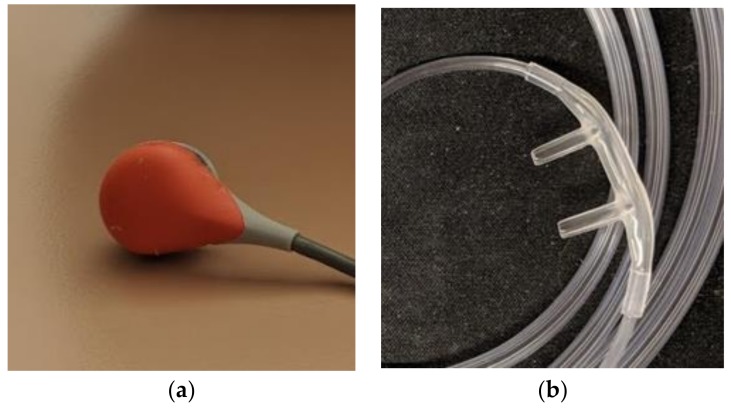
Overnight oximetry demonstrating normal baseline oxygen saturation and frequent oxygen desaturations suggestive of central sleep apnea.

Capnography refers to the continuous recording of CO_2_ measurements and can be done in isolation or in association with oximetry (i.e., oxycapnography). This can be accomplished with an end-tidal sensor, via a nasal cannula that appears similar to oxygen prongs, and/or with a transcutaneous sensor, which is placed on a membrane on the skin (see Figure 7). The recording of CO_2_ levels overnight can determine whether a child meets the criteria for nocturnal hypoventilation, much as a PSG does. However, as with oximetry, it does not allow for the differentiation between types of SDB. The British Thoracic Society clinical practice guideline for NMD suggests that all children with abnormal overnight oximetry should receive at least oxycapnography, if a full PSG is not available [3]. Performing accurate capnography can be difficult in the ambulatory setting, as these monitors are typically expensive, require calibration, and are susceptible to significant artifacts at times [34]. In addition, end-tidal monitoring may be inaccurate in those with tachypnea, mouth breathing, and/or ventilation perfusion mismatch in the lungs. Confounders of transcutaneous monitoring include signal drift and body habitus, as true CO_2_ levels are underestimated in obesity. The sensor also requires rotation of skin sites to avoid burns if being used for greater than 8 h. Carbon dioxide monitoring in most hospitals is available in the intensive care units only and is rarely available in the general inpatient wards. In North America, capnography is not readily available in-home care, which is in contrast to many parts of Europe, where it is widely used [35]. Nevertheless, capnography, usually in conjunction with oximetry, may be considered a good tool for screening for SDB in patients with NMD.

### 6.3. Polysomnography

A PSG or sleep study is considered to be the gold standard for the diagnosis of SDB. It is suggested for children with suspected SDB that (1) a definitive diagnosis be made, and (2) the severity be characterized. Polysomnograms also play a role in the therapeutic management of children with NMD, as these can be used to titrate ventilator settings in order to control respiratory events, normalize gas exchange, and improve sleep fragmentation.

Full polysomnography in children includes monitoring with electroencephalograms (EEGs), electromyograms (EMGs), electrooculograms (EOGs), electrocardiograms (ECGs), nasal pressure transducers, thermistors, end-tidal and/or transcutaneous CO_2_, oxygen saturation, chest wall and abdominal movements with inductance belts, body position, snoring, and video—overnight in a sleep laboratory attended by a sleep technologist (see Figure 6). Electroencephalograms, EMGs, and EOGs are used to score arousals by observing changes in brain-wave activity and muscle tone and to stage sleep; there are three types of non-REM sleep (N1, N2, and N3) and REM sleep. A nasal pressure transducer and thermistor allow for accurate monitoring of airflow to determine apneas (cessation of airflow) and hypopneas (reduction of airflow). Chest and abdominal belts with flow signals then allow for events to be characterized as obstructive (snoring, flattening of nasal pressure or flow signal, and/or paradoxical movements during respiratory events) or central (absence of the previously mentioned obstructive criteria), according to the AASM scoring manual [31], although the characterization of hypopneas as obstructive versus central is controversial [36].

**Figure 8:**
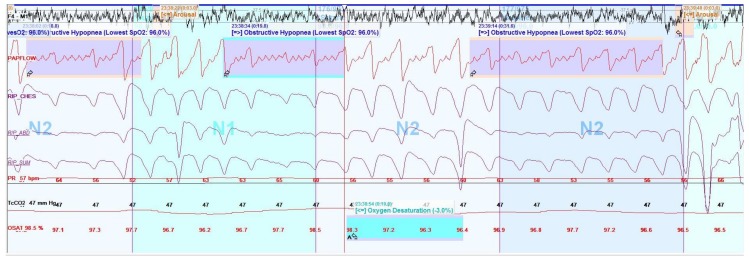
Screen shot (2 min) of selected information derived from a polysomnogram montage demonstrating obstructive hypopneas (smaller deflections in flow signal are cardiac oscillations, not flow). F4-M1 represents electroencephalogram (EEG) signal; PAPFLOW represents flow signal from non-invasive positive pressure therapy sensor; RIP-CHES, RIP-ABD, and RIP-SUM represent inductance belt signals; Tc CO_2_ represents transcutaneous sensor reading; OSAT represents oxygen saturation reading.

Typically, sleep studies in children are performed over an 8–10 h period of nocturnal sleep. A minimum of 4 h of sleep is the generally accepted standard for a valid study result, although this has been debated. Studies are then scored for arousals, respiratory events, desaturations, periodic limb movements, and time spent at various oxygen saturations and CO_2_ levels, during various stages of sleep, to allow for diagnosis (see Figure 8). Relevant parameters to report include the obstructive apnea-hypopnea index (OAHI), the central apnea-hypopnea index (CAHI), total sleep time spent with a CO_2_ level above 50 mmHg, the desaturation index, and the arousal index. The OAHI is the number of obstructive events divided by the total sleep time, and the CAHI is the same but for central events, both expressed in events per hour. These indices guide diagnosis and management decisions and are adjuncts to clinical history, physical examination, and pulmonary function tests in children with NMD. Management options may include adenotonsillectomy, other airway surgeries (e.g., lingual tonsillectomy), or non-invasive positive pressure therapy, depending on the clinical picture.

The sleep montage setup for PSG has several leads that are connected to the child, which can be difficult for the child to tolerate and can lead to failure to capture sleep adequately. This can lead to an unrepresentative sample of sleep captured, often called “the first night effect,” in which more sleep fragmentation and atypical percentages of various sleep stages may be observed. To address this, there has been an attempt to develop home monitoring devices of similar quality to PSG; these home PSG devices have been validated in adults, but none are yet approved for children.

There are additional challenges in performing PSGs in children with NMD. These include a potential lack of wheelchair accessibility, safe transfer/lifting systems, necessary specialized beds/equipment, accessible shower and bathroom facilities, and trained personnel required for routine turning throughout the night. Furthermore, pediatric PSG is often less readily available, with long waiting lists due to the resources and expertise required to perform this test [37].

The indications to perform a sleep evaluation in children with NMD vary between guidelines and specific conditions. The risk of nocturnal hypoventilation has been shown to increase as FEV_1_ declines in children with DMD [38]. Recent American guidelines for patients with Spinal Muscular Atrophy (SMA) separate children into “non-sitters,” “sitters,” and “walkers”. Sleep studies are routinely recommended for the first two groups, whereas clinical evaluation and sleep study are only recommended in the presence of clinical symptoms in “walkers”. [39] The low threshold for performing sleep studies is due to the fact that a negative symptom report and reassuring spirometry may not be predictive of the absence of SDB [40]. The British Thoracic Society guidelines recommend sleep assessments in patients with clinical symptoms suggestive of SDB who have lost ambulation, who will never gain ambulation, or who are infants with NMD [3]. Canadian Thoracic Society guidelines recommend evaluation for SDB if the FVC in spirometry is less than 60% predicted, in those who are symptomatic, or in those who have become non-ambulant because of muscle weakness on at least a yearly basis [41]. The recommendations for which test to use are often contentious but typically take a pragmatic approach, with PSG being the ideal but oximetry or oxycapnography being reasonable alternatives for initial testing if PSG is unavailable. The frequency of screening is also variable between guidelines, with most recommending no less than yearly screening for those at risk of SDB. Sleep studies are also indicated for those starting or being maintained on non-invasive positive pressure therapy; this is often done on a yearly basis or sooner if there is significant change in the child’s clinical status to ensure the ventilator settings are appropriate.

## 7. Conclusions

Children with NMD are at a high risk of developing respiratory complications, including atelectasis, pneumonias, and respiratory insufficiency [1,2]. Respiratory diagnostic tests are important tools to help to evaluate the respiratory status at the time of diagnosis and aid in prognosis, as well as to monitor disease progression [4].

A restrictive pattern is commonly evident in the pulmonary function tests of individuals with NMD [4]. Specific respiratory muscle testing is usually decreased in comparison to healthy children [20]. On the basis of the existing literature, certain threshold values have been identified to guide clinicians in deciding when to initiate further treatments, including cough augmentation therapy, or further testing, such as a sleep evaluation (see Table 2).

As individuals with NMD become weaker, the risk of SDB increases [38]. Polysomnograms are considered the gold standard for evaluating SDB; however, oximetry, capnography, and particularly oxycapnography are considered reasonable alternatives when formal sleep studies are unavailable.

Ongoing respiratory evaluations, both clinical and diagnostic, are crucial in children with NMD. A clinical evaluation of respiratory health should be included in every medical consultation for children with NMD [3]. Visits to a physician specializing in pediatric respiratory care should occur every 3–6 months for children deemed at a higher risk of respiratory complications (e.g., aged >12 years, falling in below normal range of FVC, and non-ambulatory), with pulmonary function testing, respiratory muscle strength testing, and evaluation of PCF organized with each visit [10]. An assessment of SDB should occur no less than annually for children with a VC of <60% predicted who have become non-ambulant or who will never attain the ability to walk—and earlier in those with new sleep symptoms or abnormal daytime gas exchange [3].

## Figures and Tables

**Table 1:** Restrictive pattern of pulmonary function testing (PFT) in children characteristic of neuromuscular disease (NMD).

Test	PFT Findings
FEV_1_	↓
FEV_1_/FVC	Normal
FVC or VC	↓
TLC	↓
RV	↑
FRC	Normal
MIP	↓
MEP	↓
PCF	↓

FEV_1_, volume of air exhaled forcefully; FVC, total volume of air forcefully expelled; VC, volume capacity; TLC, total lung capacity; RV, residual volume; FRC, functional residual capacity; MIP, maximal inspiratory pressure; MEP, maximal expiratory pressure; PCF, peak cough flow.

**Table 2:** Clinical management recommendations for children with neuromuscular disease (NMD) on the basis of diagnostic test results.

Recommendation	Diagnostic Test Results
Initiate cough augmentation device	PCF < 270 L/min for children ≥12 years of age
	MEP < 60 cmH_2_O
Perform overnight sleep monitoring	FVC < 60% predicted
	MIP < 40 cmH_2_O
	Clinical symptoms suggestive of SDB
	Loss of ambulation
	Children with NMD that will never have the ability to ambulate
	Infants with NMD
Initiate nocturnal non-invasive ventilation (DMD specifically)	Baseline awake SpO_2_ < 95%
	Baseline awake pCO_2_ > 45 mmHg
	FVC < 30–50% if undergoing a surgical procedure
	Evidence of SDB on PSG

SDB—sleep-disordered breathing; DMD—Duchenne muscular dystrophy; PSG—polysomnogram
